# The Probiotic Mixture VSL#3 Accelerates Gastric Ulcer Healing by Stimulating Vascular Endothelial Growth Factor

**DOI:** 10.1371/journal.pone.0058671

**Published:** 2013-03-06

**Authors:** Poonam Dharmani, Claudio De Simone, Kris Chadee

**Affiliations:** 1 Department of Microbiology and Infectious Diseases, University of Calgary, Calgary, Alberta, Canada; 2 Department of Experimental Medicine, University of L'Aquila, L'Aquila, Italy; McMaster University, Canada

## Abstract

Studies assessing the effect and mechanism of probiotics on diseases of the upper gastrointestinal tract (GI) including gastric ulcers are limited despite extensive work and promising results of this therapeutic option for other GI diseases. In this study, we investigated the mechanisms by which the probiotic mixture VSL#3 (a mixture of eight probiotic bacteria including *Lactobacilli*, *Bifidobacteria* and *Streptococcus* species) heals acetic acid induced gastric ulcer in rats. VSL#3 was administered orally at low (6×10^9^ bacteria) or high (1.2×10^10^ bacteria) dosages from day 3 after ulcer induction for 14 consecutive days. VSL#3 treatments significantly enhanced gastric ulcer healing in a dose-dependent manner. To assess the mechanism(s) whereby VSL#3 exerted its protective effects, we quantified the gene expression of several pro-inflammatory cytokines, protein and expression of stomach mucin-Muc5ac, regulatory cytokine-IL-10, COX-2 and various growth factors. Of all the components examined, only expression and protein production of VEGF was increased 332-fold on day 7 in the ulcerated tissues of animals treated with VSL#3. Predictably, animals treated with VEGF neutralizing antibody significantly delayed gastric ulcer healing in VSL#3 treated animals. This is the first report to demonstrate high efficacy of the probiotic mixture VSL#3 in enhancing gastric ulcer healing. Probiotic efficacy was effective at higher concentrations of VSL#3 by specifically increasing the expression and production of angiogenesis promoting growth factors, primarily VEGF.

## Introduction

Gastric ulcer healing is a spontaneous, complicated array of different mechanisms that work in tandem to correct the imbalance between the aggressive (eg, acid, pepsin, proinflammatory cytokines and *Helicobacter pylori*) and defensive factors (eg, mucus, bicarbonate, blood flow and growth factors) in stomach [1]. The process involves mitigation of the aggressive luminal factors, filling of mucosal defect with proliferating and migrating epithelial cells and connective component so as to reconstruct the mucosal architecture [2]. We and others [3], [4], [5] have shown the efficacy and putative mechanism of different drugs and herbs in enhancing the process of spontaneous ulcer healing in various *in vivo* gastric ulcer models. However, studies assessing the effect of probiotics on diseases of the upper gastrointestinal tract (GI) and particularly on gastric ulcer disease are limited despite the extensive work and promising results of this therapeutic option for other GI diseases including inflammatory bowel disease [6], [7], [8], [9].

Probiotics are live microorganisms that in addition to the nutritious value bestow several health benefits when provided in ample quantity. VSL#3 is a probiotic preparation containing a mixture of eight bacterial species, four species of *Lactobacilli* (*acidophilus*, *bulgaricus*, *casei*, *plantarum*), three species of *Bifidobacteria* (*breve*, *infantis*, *longum*) and one *Streptococcus* species, which has been shown to be clinically effective in preventing flare-ups of refractory pouchitis [8] and lead to remission of ulcerative colitis [9]. We have previously shown that VSL#3 or its secreted products are potent colonic mucin secretagogues *in vitro* on human colonic epithelial cells and in colonic loop studies in animal models [10]. However, the impact of probiotic treatment on acute gastric damage associated with gastric ulcers is not well explored. The primary reason is that the gut provides a host of adverse physiological conditions like acidic pH, digestive enzymes, bile acid and mechanical stress that attenuates the survival and growth of probiotic microorganisms. However, treatment with ample quantity of multiple probiotic microorganisms may overcome this limitation. There are a few reports of individual probiotic bacteria including *Lactobacillus rhamnosus* [11], *L. gasseri* [12], *L. acidophilus* [13] and *Bifidobacterium* [14] conferring protection against gastric ulcers in promoting ulcer healing in different *in vivo* ulcer models. However, studies on the effect of probiotic bacteria on enhancing *H. pylori* eradication were inconsistent in their findings [14], [15]. A recent study has shown that adequate supplementation with mixture of 8 probiotics strains might eradicate *H. pylori* [16]. At present, it still not known whether mixed probiotic bacteria are more effective than single strains in promoting ulcer healing. In the present study, we investigated the cellular mechanisms whereby VSL#3 probiotic mixture augmented gastric ulcer healing in rats.

## Materials and Methods

### Animals

Six-week-old male Sprague–Dawley rats weighing between 150 and 200 g (Charles River, St. Constant, Quebec) were housed in cages 2 per group at a constant room temperature, with 12-h light and dark cycles, and fed standard rodent chow and water *ad libitum*. Following a 7-day acclimation period, rats were randomized into experimental and control groups for induction of gastric ulcer. The Animal Experiment Ethics Committee of the University of Calgary approved this study.

### Experimental Design

To study the effect of VSL#3 probiotic mixture on gastric ulcer healing and mitigation of offensive luminal factors (pro-inflammatory cytokines) and promotion of cytoprotective defensive factors (regulatory cytokines, mucin and growth factors), rats were divided into three experimental groups. Controls animals were treated with vehicle after ulcer induction and two other groups that received either low (6×109 bacteria/animal/day) or high (1.2×1010 bacteria/animal/day) dose VSL#3. Animals were sacrificed on day 3, 7 and 14 of vehicle or VSL#3 treatment (for rationale, see section on gastric ulcer induction and VSL#3 treatments). A total of 18 animals were utilized for each time point (*N* = 6 for each trial/time point) and the study was done in triplicate. To investigate the effect of anti-vascular endothelial growth factor (VEGF) on gastric ulcer healing, neutralizing antibodies against VEGF were used. In this experiment, four groups were used. After ulcer induction, a control group was treated with vehicle only. The treatment groups included rats treated with neutralizing VEGF antibody alone, rats that received high dose VSL#3 (1.2×1010 bacteria/animal/day) together with a control IgG antibody and rats that received high dose VSL#3 (1.2×1010 bacteria/animal/day) together with neutralizing VEGF antibodies. The antibody treated groups (IgG and neutralizing VEGF antibody) were given antibodies at a dose of 10 mg/kg BW (for rationale, see section on VEGF neutralizing antibody treatment). Animals were sacrificed by cervical dislocation on day 3, 7 and 14 following anti-VEGF treatment. A total of 24 animals were utilized for each time point (n = 6 for each trial/time point).

### Gastric Ulcer Induction and VSL#3 treatment

Chronic gastric ulcers were induced experimentally in rats as previously described [17]. Under anesthesia with pentobarbitone (35 mg/kg body weight, i.p.), the abdomen was opened by midline incision. A plastic tube of 6 mm, opened at both the ends was applied tightly to the serosal surface of anterior wall of the stomach just proximal to antral gland area. Onto the surface of stomach, 60 μl of 40% acetic acid was poured through a tube for 90 seconds. This produced immediate necrosis of the entire mucosa and sub-mucosa within the area where the acetic acid was applied. Acetic acid remaining on the surface was then wiped off with a sterile filter paper and the opened abdomen was closed. We have previously shown that this procedure produces histologically well-characterized ulcers within 3 days after acetic acid exposure and heals completely within 3 weeks without perforation or penetration to the surrounding organs [3]–[4].

Treatment of VSL#3 at low (6×10^9^ bacteria) and high dosages (1.2×10^10^ bacteria) was based on the results of preliminary studies using five different dosages of VSL#3 (1.5×10^9^, 3×10^9^, 6×10^9^, 1.2×10^10^ and 2.4×10^10^ bacteria/animal/day, respectively). VSL#3 dosages of 1.5×10^9^ and 3×10^9^ bacteria/animal/day were not found effective in promoting gastric ulcer healing while the effect of the dose of 2.4×10^10^, bacteria/animal/day on gastric ulcer healing was not significantly different from that of 1.2×10^10^ bacteria/animal/day (Figure S1). Treatment with VSL#3 was started when ulcers were fully developed on the 3rd day following acetic acid administration; this was considered as day 1 of treatment for the next 14 days. The pH of the different concentrations of VSL#3 was 7.0. Animals were sacrificed on day 3, 7 and 14 following VSL#3 treatment. The ulcerated and non-ulcerated area from each animal was rinsed with ice-cold phosphate-buffered saline placed on ice and three cross sections were collected. Two cross sections were snap-frozen in liquid nitrogen and stored at −70°C for RNA isolation and protein preparation. Third cross section was immediately fixed in 10% neutral buffered formalin for histological analysis.

### Assessment of ulcer size, percentage healing and histological examination

The total ulcer area and percentage ulcer healing at different times post treatments were assessed in rats stomach by cutting along the greater curvature and the ulcer (mm^2^) area measured. Two different individuals measured the ulcer area in a blinded fashion. The percentage healing was calculated as:
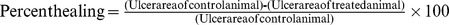



Histological studies were performed according to the previously described method [18]. Briefly, the ulcer crater of each stomach was sectioned out and fixed overnight in 10% neutral buffered formalin, then dehydrated gradually in ethanol and embedded in paraffin, using neoclear as intermediate solvent. Sections of 6 μm were obtained in an automated microtome. Ulcerated sections were stained with haematoxylin and eosin (H&E) for histological evaluation during healing. Microscopically, H&E tissues were reported as showing: a normal appearance, mild infiltrates of inflammatory cells into the lamina propria mucosa, with either no or only shallow erosion, or deep erosion and ulceration.

### Quantitative real-time PCR analysis

Quantitative real time PCR analysis was performed to assess changes in the expression of genes encoding for secretory mucin (Muc5ac), cyclooxygenase-2 (COX-2), pro-inflammatory (TNF-α and IL-1β) and regulatory (IL-10 and TGF-β) cytokines and vascular endothelial growth factor (VEGF), transforming growth factor-β (TGF-β) and epidermal growth factor (EGF). Total RNA was extracted from ulcerated and non-ulcerated tissue with TriZol reagent (Invitrogen). The yield and purity of the RNA was determined by spectroscopic analysis. 2 μg of RNA was reverse transcribed by using M-MLV reverse transcriptase (Invitrogen) as per manufacturer's instructions. One μL of cDNA was used for real-time PCR (Corbett Research). Real-time primers used with the specific annealing temperature are shown in Table 1. Amplifications were carried out with Qiagen's Quantitect SYBR Green PCR kit by using the following cycling conditions: 94°C hold for 15 min, followed by 40 cycles of denaturation at 94°C for 20 s, annealing at corresponding temperatures for 30 s and extension at 72°C for 30 s. Specificity of amplification was checked by melt curve analysis. The mRNA expression of different gene was normalized against GAPDH, and fold change over control was determined according to the ddCt method [19].

**Table 1:** Rat primer sequences and their annealing temperature.

Gene		Primer Sequence	Annealing temperature
Muc5ac	Forward	GTGCGGCACTTGCACCAACG	60°C
	Reverse	GCCCTTGGCAGGAAGGCTGG	
COX-2	Forward	GGTTCACCCGAGGACTGGGC	60°C
	Reverse	CGCAGGTGCTCAGGGACGTG	
IL-1β	Forward	CACCTCTCAAGCAGAGCACAG	59°C
	Reverse	GGGTTCCATGGTGAAGTCAAC	
TNF-α	Forward	AAATGGGCTCCCTCTCATCAGTT	59°C
	Reverse	TCTGCTTGGTGGTTTGCTACGAC	
IL-10	Forward	GGCTCAGCACTGCTATGTTGCC	65°C
	Reverse	AGCATGTGGGTCTGGCTGACTG	
VEGF	Forward	CCTCGCTCCCGGTCCATCCA	60°C
	Reverse	CGAAACCCTTGACCTCGCCCC	
TGF-β	Forward	CCTGCACAGCTCCAGGCACC	60°C
	Reverse	TGCTCCACCTTGGGCTTGCG	
EGF	Forward	GCACCAACACGGAGGGAGGC	60°C
	Reverse	GTACGACGGCGGGCATCCTG	
GAPDH	Forward	TGACAACTCCCTCAAGATTGTCA	60°C
	Reverse	GGCATGGACTGTGGTCATGA	

Muc: Mucin; COX: Cyclooxygenase; IL: Interleukin; TNF: Tumor necrosis factor; TGF: Transforming growth factor; EGF: Epithelial Growth factor; GAPDH: Glyceraldehyde-3-phosphate dehydrogenase.

### Protein Levels of VEGF, TGFβ and EGF in gastric tissue homogenate

Gastric tissues were homogenized in cold phosphate buffer saline (PBS, pH 7.4) using a Polytron homogenizer. The tissue homogenate was then centrifuged at 20,000× g for 20 min at 4°C to obtain the clear supernatant and protein concentrations measured using a BCA (Bicinchoninic acid) kit. The protein levels of VEGF, TGF-β and EGF in homogenized gastric tissues from both the ulcerated and non-ulcerated mucosa were measured using ELISA kits according to the manufacturer's protocol (rat VEGF ELISA Kit, Ray Biotech; rat TGF-β kit, ebioscience and rat EGF ELISA kit, Uscn Life Science Inc.). VEGF, TGF-β and EGF level was expressed as pg/mg of tissue.

### The effect of neutralizing anti-VEGF antibody on gastric ulcer healing induced by VSL#3

To evaluate the effect of neutralizing anti-VEGF antibody on gastric ulcer healing promoted by VSL#3, four groups of animals were used namely, control, neutralizing VEGF antibody (R&D systems), VSL#3 high dose + IgG (isoform antibody, R&D systems), and VSL#3 high dose + neutralizing VEGF antibody (R&D systems). In preliminary experiments, three doses of neutralizing VEGF antibody (5, 10 and 20 μg/animal/day; ip) were tested in animals receiving a high dosage of VSL#3 for 14 consecutive days and a dosage of 10 μg/animal/day was found to significantly reducing healing and was selected for use for all subsequent experiments. Animals were sacrificed on day 3, 7 and 14 days after VSL#3 + antibody treatment. Gastric ulcer area was measured and histological examination, quantitative real time PCR analysis and ELISA based protein analysis was performed as described above.

### Statistical analysis

Results are expressed as means ± SEM. Significance differences between control and VSL#3 treated groups were determined using Kruskal-Wallis test with Dunns post-test to compare specific groups. The choice of a non-parametric test (Kruskal-Wallis test) instead of a parametric test (Analysis of Variance, ANOVA) was based on the fact that at least one of the groups in all but three of the comparisons was non-Gaussian. To maintain consistency, Kruskal-Wallis test was used for all comparisons. All statistical analyses were performed using Graph Pad Instat software. A *P* value of >0.05 was considered significant.

## Results

### VSL#3 probiotic mixture accelerates gastric ulcer healing

**Figure 1:**
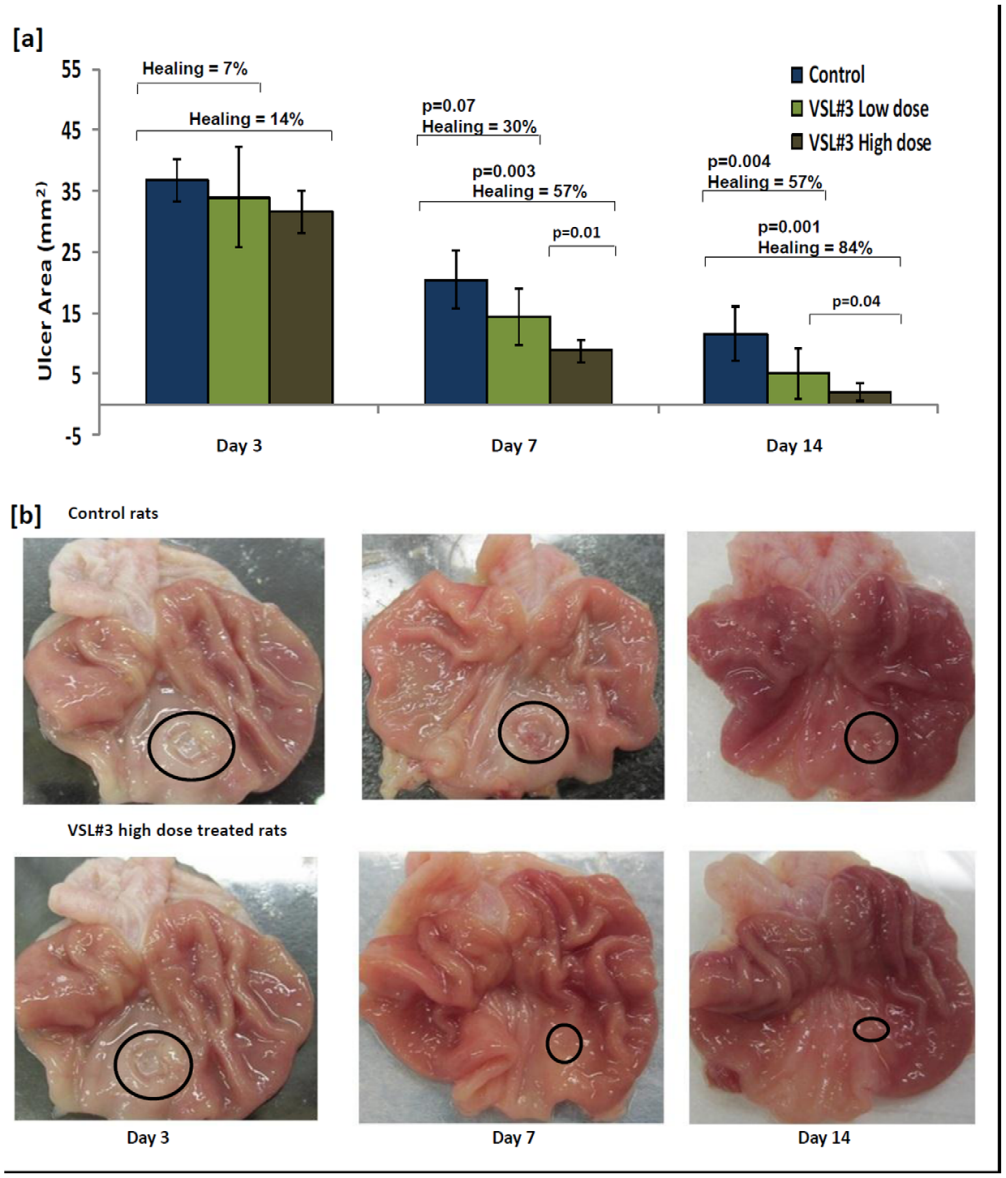
Effect of VSL#3 treatment on acetic acid induced gastric ulcer healing in rats. [**a**] The ulcer area (mm^2^) plotted for animals with acetic acid induced gastric ulcer on day 3, 7 and 14 of treatment with vehicle (Blue), VSL#3 low (light green) and high (dark green) dose. Data is represented as means ± SEM from 6 animals per day. Percent ulcer healing calculated for animals treated with VSL#3 low (light green) and high (dark green) dose in comparison to the control animals as well as the significant p values are represented in the upper section of the plot. [**b**] Morphological view of acetic acid induced gastric ulcer on day 3, 7 and 14 of treatment with vehicle (Top), VSL#3 high dose (Bottom).

Following serosal application of acetic acid 100% of animals developed gastric ulcers after 3 days. Thereafter, the gastric ulcers progressively healed up to day 14 (Fig. 1a). To determine whether the probiotic mixture VSL#3 could accelerate gastric ulcer healing, animals were treated with either low or high dosages of VSL#3 starting on day 3 when ulcers were fully developed for the next 14 consecutive days. As shown in Figure 1 a/b, ulcer healing on day 3 of treatment was not significant in either the control or VSL#3 treated groups. Thereafter, there was a progressed increase in ulcer healing reflected by a decrease in the ulcer area on day 7 in animals treated with either low (p = 0.07) or high (p = 0.003) dosages of VSL#3 as compared to untreated controls (Fig. 1a). In particular, VSL#3 at high dosages for 14 days led to a complete ulcer healing in ∼50% of animals (Fig. 1 a), with no sign of ulcerated mucosa. In the remaining animals there was significant restoration of the surface epithelium and extensive tissue regeneration (Fig. 1b). While VSL#3 conferred significant increase in ulcer healing, high dose treatment (% healing was 57% and 84% on day 7 and 14, respectively) significantly outperformed low dose treatments (% healing was 30% and 57% on day 7 and 14, respectively; Fig. 1a). Since, day 3 of treatment did not show significant differences in ulcer healing for control and VSL#3 treated groups, all results in the subsequent sections are shown for days 7 and 14 of treatment.

### VSL#3 treatment promotes tissue repair in the ulcerated gastric mucosa

**Figure 2:**
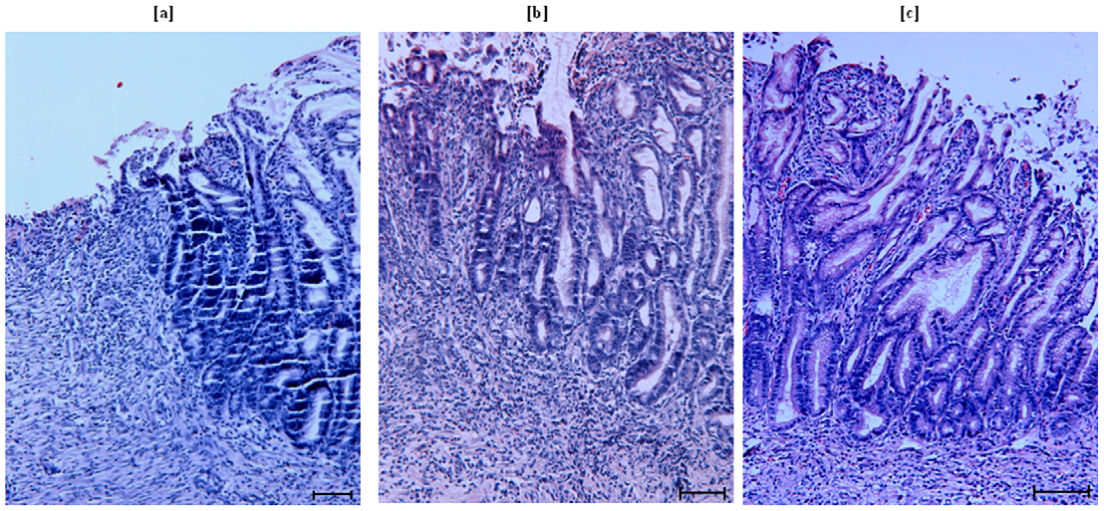
Histological alterations in the ulcerated mucosa of control and VSL#3 treated rats. Sections of ulcerated stomach obtained from rats of control and VSL#3 low and high dose treated groups in acetic acid induced chronic ulcer model after 14 days of treatment (*n* = 6 in each group). Scale bar represents 25 µm. [**a**] Sections of ulcerated stomach obtained from control group rats: minimal repair with inflammatory exudates were seen. [**b**] Sections of ulcerated stomach obtained from VSL#3 low dose treated group: Superficial remodeling with in-complete re-epithelization of the mucosa and starting of gland organization was seen. [**c**] Sections of ulcerated stomach obtained from VSL#3 high dose treated group: Almost complete restoration of the mucosal epithelium with organized gland was seen.

H&E staining revealed different histological signs of healing among the different groups. On day 14, similar to our gross finding in ulcer area measurement, control rats showed minimal repair. There were reduced inflammatory exudates, minimal extent of mucosal regeneration with reduced size of the ulcer crater. We did not observe much glandular organization in control rats (Fig. 2a). However, in animals treated with high dose VSL#3 that showed maximum healing in reduction on the size of the ulcer area, there was restoration of the mucosal epithelium (re-epithelization) and few inflammatory cells. As shown in Figure 2c, secretory glands were also arranged. Assessment of the ulcerated tissues in VSL#3 low dose treated group showed some superficial remodeling with modest inflammatory exudates and in-complete re-epithelization of the mucosa. Proper organization of glands was also seen at some places (Fig. 2b).

### Effect of VSL#3 treatment on Muc5ac, COX-1/2 and pro-inflammatory and regulatory cytokine gene expression

The mucus layer is the first defensive layer of the gastric mucosa [20] that plays a major protective role against acid and pepsin in the stomach. Since Muc5ac is the most abundantly expressed mucin in the normal gastric mucosa, we determine if VSL#3 treatments affected Muc5ac gene expression in the ulcerated and/or non-ulcerated tissues. As shown in Table 2, even though Muc5ac expression was modestly increased in VSL#3 treated animals, it was not significantly different among the groups tested. Prostaglandins are the other important defensive factor critical for the maintenance of gastric mucosal integrity, whose synthesis is regulated by the rate limiting enzyme-cyclooxygenase (COX), which exists in two isoforms-COX-1 and COX-2 [21]. Constitutively expressed COX-1 was also found to be unchanged in the ulcerated and non-ulcerated tissues whereas the expression of COX-2 was significantly higher in the VSL#3 high dose treated group on day 7 but not on day 14 (Table 2).

**Table 2:** Change in the expression of genes encoding Muc5ac, COX2, TNF-α, IL-1β and IL-10 in the ulcerated and non-ulcerated regions of VSL#3 treated animals.

Gene	Groups	Day 7		Day 14	
		Fold increase in gene expression a	P value b	Fold increase in gene expression a	P value b
Muc5ac	Control	1.2±0.3		1.5±0.2	
	VSL#3 low dose	1.8±0.5	ns	2.4±0.4	ns
	VSL#3 high dose	2.1±0.5	ns	1.9±0.4	ns
COX-2	Control	9.5±2.2		18.2±2.4	
	VSL#3 low dose	16.4±3.8	ns	22.1±2.2	ns
	VSL#3 high dose	21.8±5.1	0.05	16.4±2.1	ns
TNF-α	Control	8.9±3.2		4.9±1.0	
	VSL#3 low dose	5.8±1.8	ns	4.0±0.8	ns
	VSL#3 high dose	3.1±1.2	0.05	2.2±0.5	ns
IL-1β	Control	4.5±1.0		3.6±0.6	
	VSL#3 low dose	3.2±0.8	ns	2.5±0.5	ns
	VSL#3 high dose	3±0.8	ns	2.8±0.5	ns
IL-10	Control	7.1±1.5		13.8±2.1	
	VSL#3 low dose	5.2±1.8	ns	7.4±1.5	ns
	VSL#3 high dose	3.8±0.5	ns	8.6±1.8	ns

a Gene expression is shown as fold change in ulcerated tissue as compared to non ulcerated tissues. Data shown are the means ± SEM for 6 animals/time point.

b P values were calculated for VSL#3 treated group with controls as the reference group.

The pro-inflammatory cytokines TNF-α and IL-1β have been shown to play an important role in the pathogenesis of gastric ulcer by initiating an early inflammatory process [22], while the regulatory cytokine IL-10 is known to attenuate TNF-α production [23]. Expression of both TNF-α and IL-1β genes was found to be elevated in the ulcerated tissues in the three groups of animals. However, VSL#3 treatment did not affect the expression of IL-1β and IL-10 and marginally decreased the expression of TNF-α in the ulcerated tissue when compared with the vehicle treatment (Table 2).

### VSL#3 treatment stimulates the production of growth factors

**Figure 3:**
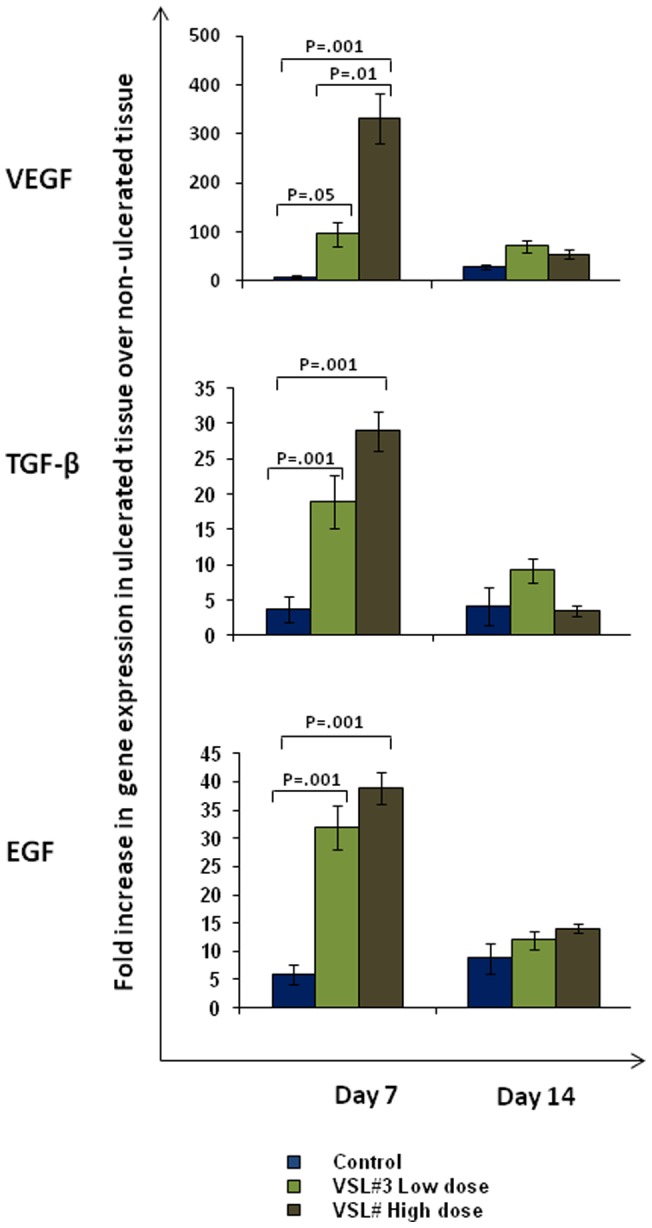
Effect of VSL#3 treatment on gene expression of different growth factors in rats with acetic acid induced gastric ulcers. The relative gene expression levels determined by real time PCR for VEGF [Top], TGF-β [Middle] and EGF [Bottom] using mRNA extracted from control (Blue), VSL#3 low (light green) and high (dark green) dose treated animals on day 7 and day 14 of treatment. Expression levels of all genes were normalized using GAPDH as housekeeping gene. The mRNA expression is graphed as fold change in ulcerated tissue over non-ulcerated tissue. Data shown are the means ± SEM of 6 animals/day. P values for all significant comparison ( p<0.05) are represented.

**Figure 4:**
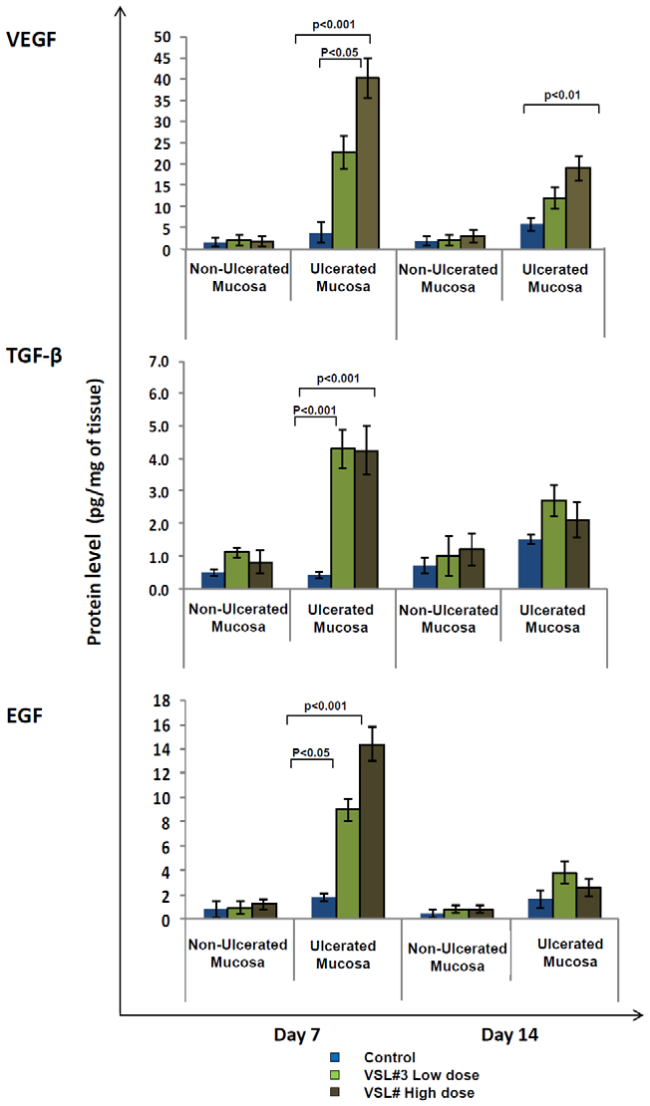
Effect of VSL#3 treatment on protein production of different growth factors in rats with acetic acid induced gastric ulcers. Protein levels in tissue homogenates was measured by ELISA for VEGF [Top], TGF-β [Middle] and EGF [Bottom] in control (Blue), VSL#3 low (light green) and high (dark green) dose treated animals on days 7 and 14 of treatment. Protein levels in all groups are represented as pg/mg of tissue. Data shown are the means ± SEM of 6 animals/day. P values for all significant comparison ( p<0.05) are represented.

The role of growth factors VEGF, TGF-β and EGF in angiogenesis and epithelial cell migration and proliferation in gastric ulcer healing is well documented [24], [25]. As shown in Figure 3, there was a significant increase in VEGF (P<0.001) gene expression in the ulcerated tissues on day 7 in animals treated with low or high doses of VSL#3 as compared to animals treated with vehicle only (97 and 332 fold increase in gene expression in VLS#3 low and high dose treated rats, respectively). The increase in VEGF gene expression was also evident on day 14 and was most prominently in low dose VSL#3 treated rats (Fig. 3, top). VEGF expression was not altered in the non-ulcerated tissues of all the groups examined. VEGF protein content in the gastric tissue homogenate corroborated the findings of the VEGF gene expression data (Fig. 4, top). Similarly, TGF-β and EGF (Fig. 3 middle and bottom panels) gene and protein levels (Fig. 4 middle and bottom panels) were significantly increased in VSL#3 treated animals, though the change was less pronounced to that observed for VEGF.

### VEGF neutralizing antibody attenuates VSL#3-mediated gastric ulcer healing

**Figure 5:**
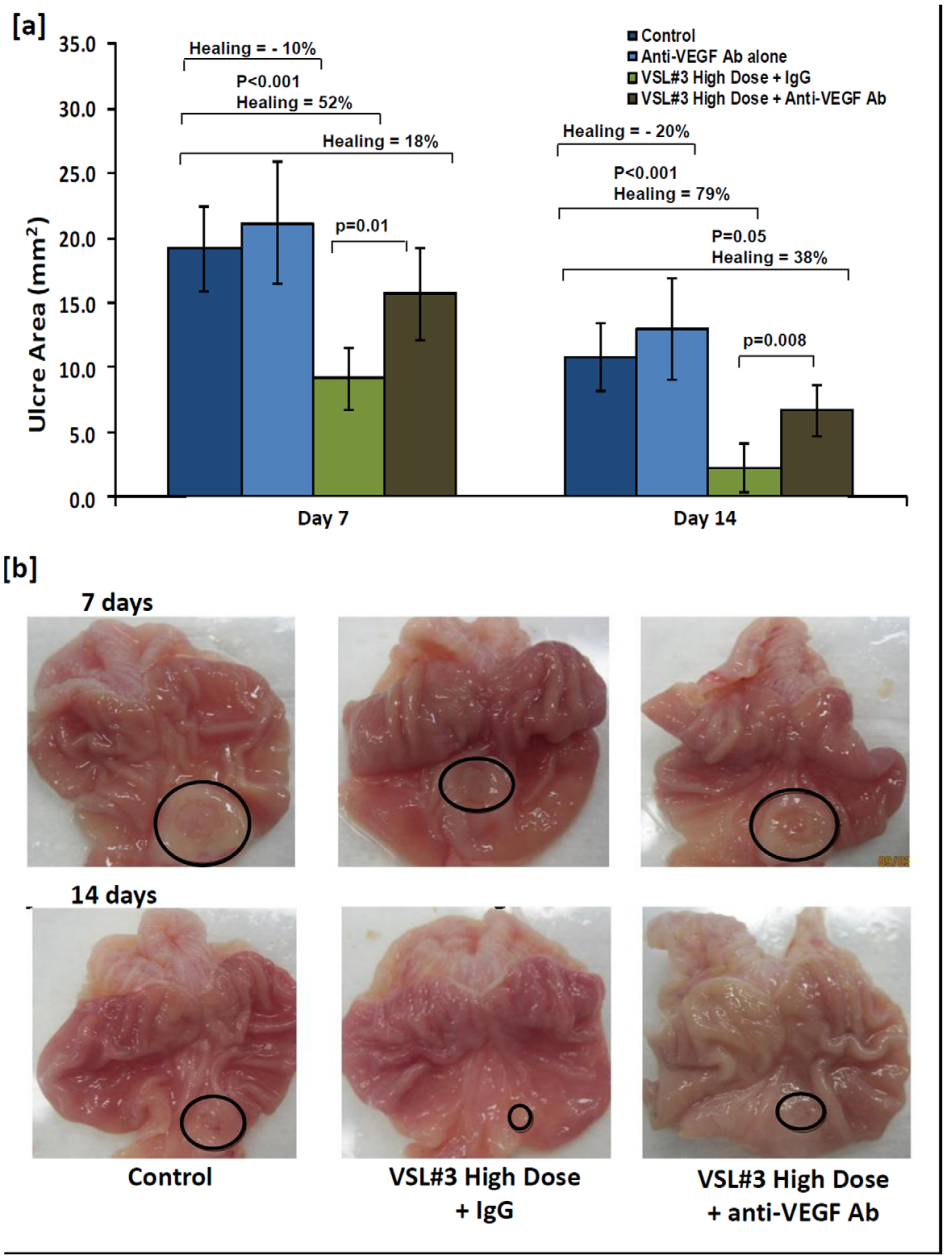
Effect of anti-VEGF neutralizing antibody treatment on acetic acid induced gastric ulcer healing in rats. [**a**] The ulcer area (mm^2^) plotted for animals with acetic acid induced gastric ulcer on days 7 and 14 of treatment with vehicle (Dark blue), VEGF neutralizing antibody alone (light blue), VSL#3 high dose + IgG (light green) and VSL#3 high dose + anti-VEGF neutralizing antibody (dark green). Data are represented as means ± SEM from 6 animals per day. Percent ulcer healing was calculated for animals treated with VEGF neutralizing antibody (light blue), VSL#3 high dose + IgG (light green) and VSL#3 high dose + anti-VEGF neutralizing antibody (dark green) in comparison to controls (dark blue). Significant p values are represented in the upper section of the plot. The percentage healing shown for the group treated with anti-VEGF antibody alone on Days 7 and 14 represent reduction in ulcer healing when compared to untreated controls. [**b**] Morphological view of acetic acid induced gastric ulcer on days 7 [Top] and 14 [Bottom] of treatment with vehicle (left), VSL#3 high dose + IgG (middle) and VSL#3 high dose + anti-VEGF neutralizing antibody (right).

Based on the data in Figure 3 where VEGF was significantly increased in response to VSL#3 treatment that enhanced gastric ulcer healing (Fig. 1), we speculated that VSL#3 protective effect and mechanism of action was dependent on VEGF. To investigate this, animals were treated with high dose VSL#3 + VEGF neutralizing antibody. As shown in Figure 5, animals treated with VEGF neutralizing antibody decreased ulcer healing by 34% and 41% respectively, on days 7 and 14 as compared to animals treated with high dose VSL#3 + IgG or vehicle control (Fig. 5a). Gross pathology (Fig. 5b) showed that the mucosal architecture was less intact in the VSL#3 + VEGF neutralizing antibody treated rats than VSL#3 + IgG treated rats on days 7 and 14 of treatment. Animals treated with VEGF neutralizing antibody alone showed reduction in ulcer healing in comparison to control animals, however, the results were not statistically significant (Fig 5a).

## Discussion

This is the first comprehensive study to demonstrate that the probiotic mixture VSL#3 can augment healing of acetic acid induced gastric ulcers. Our results showed that VSL#3 acted in a dose-dependent manner to increase in the expression and production of VEGF, a growth factor known to promote angiogenesis and healing. VEGF was critically important in gastric ulcer healing as treatment with VEGF neutralizing antibody significantly delayed the healing process in VSL#3 treated animals.

The preventative and therapeutic role of probiotics is well reported not only in case of ulcerative colitis and Crohn's disease [8]–[9], but also in conditions as varied as burned wounds and gunshot wounds [26]–[27]. However, there are limited number of studies assessing the impact of probiotics treatment on gastric ulcers primarily due to the adverse physiological conditions of stomach that diminishes the survival and growth of microorganisms. Curiously, one study [28] demonstrated that bacterial colonization occurs rapidly after ulcer induction and in particular, colonization of certain bacteria including *Lactobacillus* could significantly improves gastric ulcer healing. Other studies have shown that probiotic bacteria *Lactobacillus* species, *Saccharomyces boulardii*, *Escherichia coli* strain Nissle 1917 are effective in preventing and treating acute gastric damage associated with acetic acid [12], stress [13], aspirin [29], ethanol [30], ibuprofen [31] and indomethacin [32] induced gastric ulcers. In humans, circumstantial evidence suggests that regular ingestion of a *Lactobacillus*-containing product protects the integrity of the gastric mucosal barrier against indomethacin [32], while others [15] have reported that the effect of using probiotic bacteria along with triple therapy is no better than using triple therapy alone for treatment of gastric ulcers. A recent report [13] suggests a novel symbiotic approach for treatment where a probiotic (*L. acidophilus*) and ginger extract were simultaneously and individually encapsulated/immobilized in alginate floating beads significantly influenced gastric ulcer healing in rats. An alternate approach is to use different quantity of multi strain probiotics for promoting ulcer healing as IBD studies have shown that multi strain probiotics are more effective than monostrain probiotics [33]. Our results confirm these findings using VSL#3, a mixture of eight bacterial species that was highly effective in enhancing epithelial cell migration and proliferation, angiogenesis, and production of granulation tissues leading to faster healing of gastric ulcers.

To address the mechanisms whereby VSL#3 accelerated gastric ulcer healing we quantifying offensive luminal and/or promoting defensive factors. Previous studies have shown that probiotics exert diverse mechanisms, including stabilization of mucosal mast cell degranulation [29], induction of sIgA production [29], reduction in the cell apoptosis to cell proliferation ratio in the stomach [11], increase transepithelial resistance [34] and promoting angiogenesis [11] to mediate gastric ulcer healing. Here, we show that VSL#3 promoted gastric ulcer healing by enhancing the expression of growth factors, mainly VEGF by several folds. VEGF is a fundamental angiogenic factor, which stimulates formation of granulation tissue and new micro vessels via angiogenesis [24] that in turn accelerates gastric and duodenal ulcer healing [35]. To date, there is only one report [11] that show *L. rhamnosus* GG can up-regulate VEGF protein during ulcer healing. In our studies, we found that on day 7 of treatment with high dose VSL#3, VEGF expression was increased 332-fold as compared to control animals. This increase was not seen as prominently on day 14 of the treatment, primarily because the majority of ulcers were already healed by that time in VSL#3-treated rats. In comparison, low dose VSL#3 treated animals continued to show high expression of VEGF on both days 7 and 14 as the ulcers were still being healed. Terminating VSL#3-induced gastric ulcer healing by treating animals with anti-VEGF neutralizing antibody confirmed that VSL#3 augmented gastric ulcer healing via a VEGF-mediated mechanism. The observation of reduced ulcer healing in animals treated with VEGF neutralizing antibody alone in comparison to controls also support a role for VEGF in spontaneous gastric ulcer healing [35]. Importantly, even though VEGF is known to be up regulated by COX-2 derived PGE_2_ [36], we found marginal changes in the expression of COX-2 in VSL#3 treated rats suggesting that VSL#3 induced VEGF expression are not COX-2 mediated.

We also found that the growth factors EGF and TGF-β were significantly up regulated in the ulcerated tissues of VSL#3 treated rats. TGF-β is known to affect extracellular matrix that turns the process of epithelial cell reconstitution on and off, while EGF promotes both epithelial restitution and proliferation in the GI tract [25]. Interestingly, VSL#3 was found to be effective only in promoting growth factors and not the defensive factors Muc5A and Cox2. It is still possible that VSL#3 might affect other defensive factors not analyzed in the present study. We have previously shown that VSL#3 treatment enhances colonic Muc2/MUC2 mucin secretion in rat colon and in human colonic epithelial cells. VSL#3 treatment in animals also did not markedly reduce the expression of the pro-inflammatory cytokines, IL-1β and to a lesser extent TNF-α. Both of these cytokines play a critical role in the formation of gastric ulcers by initiating an early acute inflammatory response [22]. These findings corroborate a recent report that assessed 13 probiotic strains on aspirin-induced gastric ulcer healing [29]. At present, we do not know which bacterial specie(s) or combinations in the VSL#3 probiotic mixture are responsible for gastric ulcer healing. Previous studies have suggested that multi strain probiotics (more specifically, multi genus strains) are more effective than mono-strain probiotics [33]. It is also possible that VSL#3 mixture being a neutral solution (different concentrations of VSL#3 have a pH of 7.0) might affect gastric acid healing by diluting the acidity of the gastric juice. However, the data presented in this study cannot infer this fact, as gastric juice acidity was not measured. Nevertheless, VSL#3 would only be having an acid diluting and not acid neutralizing effect on gastric juice, if any. In all likelihood, the acid diluting effect of VSL#3 would only contribute a secondary role to its strong cytoprotective function demonstrated by extensive up-regulation of VEGF. As VSL#3 was administered as a live cell suspension, this formulation could have exerted its protective effects as live microbes, by their metabolites, cell wall components and/or DNA content or all of the above. We have not performed an experiment to compare the effect of live microbes and heat inactivated probiotic mixture to assess this question even though previous studies have shown that heat-killed or radiation inactivated probiotic bacteria (in a dose similar to that of live microbes) failed to promote ulcer-healing [11], [12]. Further, since we did not assessed angiogenesis, the inference that VSL#3 is promoting gastric ulcer healing through angiogenesis is only speculated based on VSL#3 ability to induce a robust up regulation of VEGF, which is critically important in angiogenesis [35].

In conclusion, our studies demonstrate that VSL#3 probiotic mixture is highly effective in augmenting gastric ulcer healing. The effect is dominant at high concentrations of VSL#3 mixture and is achieved mechanistically by stimulating the expression and secretion of angiogenesis promoting growth factors, primarily VEGF. This study establishes the prospect of using VSL#3 probiotic mixture therapeutically to augment gastric ulcer healing.

## Supporting Information

Figure S1 **Effect of treatment of different concentrations of VSL#3 on acetic acid induced gastric ulcer healing in rats.** [**a**] The ulcer area (mm^2^) plotted for animals with acetic acid induced gastric ulcer on day 3, 7 and 14 of treatment with vehicle (Dark blue), VSL#3 1.5×10^9^ bacteria/animal/day (light yellow), VSL#3 3.0×10^9^ bacteria/animal/day (dark yellow), VSL#3 6.0×10^9^ bacteria/animal/day (light green), VSL#3 1.2×10^10^ bacteria/animal/day (dark green), VSL#3 2.4×10^10^ bacteria/animal/day (light blue). Data are represented as means ± SEM from 6 animals per day. Significant p values are represented in the upper section of the plot.
(TIFF)
