# Correction: The Probiotic Mixture VSL#3 Accelerates Gastric Ulcer Healing by Stimulating Vascular Endothelial Growth Factor

**DOI:** 10.1371/journal.pone.0275508

**Published:** 2022-09-27

**Authors:** Poonam Dharmani, Claudio De Simone, Kris Chadee

The Competing Interests statement for this article [1] is incomplete. The correct statement is: The authors have the following interest. At the time of publication of this study, the probiotic formulation sold as VSL#3 was produced by Dupont/Danisco in the US. Claudio De Simone (CDS) is the inventor of this probiotic formulation (also known as the De Simone Formulation). CDS is named as an inventor and assignee on patent “Dietary and pharmaceutical compositions containing lyophilized lactic bacteria, their preparation and use” (U.S. Patent No. 5,716,615) and the sole owner of the know-how used to formulate the product. At the time of publication of the article, CDS owned one share of stock in, and served as Chief Executive Officer and Director for, VSL Pharmaceuticals, Inc., which sold the De Simone Formulation until 2016. PD was funded by a Canadian Association of Gastroenterology-Axcan Pharma-CIHR Research and Fellowship Award. This does not alter the authors’ adherence to all the PLOS ONE policies on sharing data and materials.

The probiotic formulation utilized in the study was produced by Dupont/Danisco in the US.
