# Supplementary figures and images for: The Probiotic Mixture VSL#3 Accelerates Gastric Ulcer Healing by Stimulating Vascular Endothelial Growth Factor
Source: PLoS One. 2013 Mar 6;8(3):e58671. doi: 10.1371/journal.pone.0058671 (PMC3590171; doi:10.1371/journal.pone.0058671)

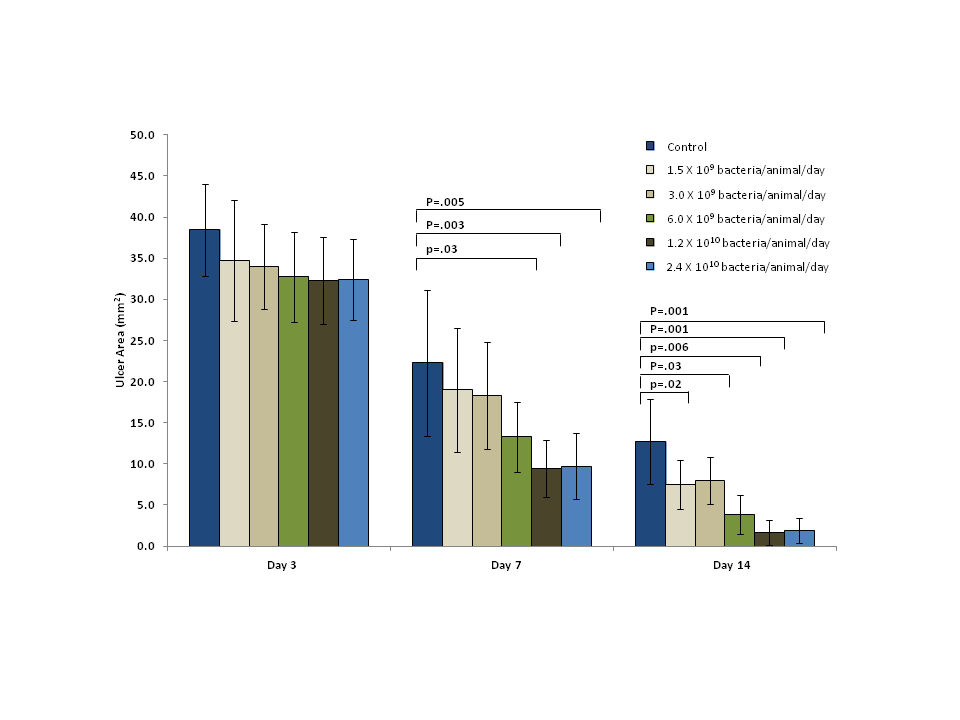

Supplement: Figure S1 — Effect of treatment of different concentrations of VSL#3 on acetic acid induced gastric ulcer healing in rats. [a] The ulcer area (mm2) plotted for animals with acetic acid induced gastric ulcer on day 3, 7 and 14 of treatment with vehicle (Dark blue), VSL#3 1.5×10^9^ bacteria/animal/day (light yellow), VSL#3 3.0×10^9^ bacteria/animal/day (dark yellow), VSL#3 6.0×10^9^ bacteria/animal/day (light green), VSL#3 1.2×10^10^ bacteria/animal/day (dark green), VSL#3 2.4×10^10^ bacteria/animal/day (light blue). Data are represented as means ± SEM from 6 animals per day. Significant p values are represented in the upper section of the plot. (TIFF) [file pone.0058671.s001.tiff]
